# Knowledge of diabetes among Gambian adults: evidence from a nation-wide survey

**DOI:** 10.1186/s12872-022-02591-z

**Published:** 2022-04-02

**Authors:** Owen Nkoka, Peter A. M. Ntenda, Yohane V. A. Phiri, Gugulethu N. Mabuza, Sihle A. Dlamini

**Affiliations:** 1 Institute for Health Research and Communication (IHRC), P.O Box 1958, Lilongwe, Malawi; 2 Institute of Health and Wellbeing, University of Glasgow, Glasgow, UK; 3 MAC-Communicable Disease Action Centre (MAC-CDAC), Kamuzu University of Health Sciences (KUHeS), Private Bag 360, Chichiri, Blantyre 3, Malawi; 4 School of Public Health, College of Public Health, Taipei Medical University, Taipei, Taiwan; 5 Ministry of Health, Eswatini Government, Mbabane, Eswatini; 6 Eswatini Nazarene Health Institutions, Manzini, Eswatini

**Keywords:** Diabetes, Knowledge, Blood glucose, Cardiovascular diseases, The Gambia

## Abstract

**Background:**

Diabetes is increasingly becoming a public health problem in developing countries like The Gambia. Prevention of diabetes and appropriate management of the disease largely depends on correct knowledge of the risk factors and signs and symptoms of the condition. However, studies that have assessed knowledge of diabetes at population level are limited. We examined the knowledge of diabetes risk factors, and signs and symptoms among Gambian adults.

**Methods:**

The 2019–2020 Gambia demographic and health survey data was used to analyze 4, 436 men and 6, 186 women. Knowledge of diabetes was assessed two-fold: (1) diabetes risk factors and (2) diabetes signs and symptoms. Several sociodemographic factors were considered for analysis. A generalized estimating equation model was fitted to test the association between the selected sociodemographic factors and diabetes knowledge.

**Results:**

Among the men, 7.6% and 3.1% had knowledge about diabetes risk factors, and signs and symptoms, respectively. Approximately 3.1% and 1.2% of the women included in the analysis had knowledge of diabetes risk factors, and signs and symptoms, respectively. Men who were aged ≥ 35 years were more likely to have knowledge regarding diabetes risk factors (adjusted odds ratio (AOR) = 1.90, 95% confidence interval (CI) = 1.12–3.22), and signs and symptoms (AOR = 2.59, 95% CI = 1.08–6.17). Having access to media was associated with increased odds of having knowledge regarding diabetes risk factors (AOR = 1.61, 95% CI = 1.09–2.37) and signs and symptoms (AOR = 2.04, 95% CI = 1.07–3.88) among men. Among other factors, educational level was positively associated with having diabetes knowledge among both men and women. Heterogeneities regarding diabetes knowledge were observed among different regions and areas of residence.

**Conclusion:**

There is a need to improve awareness regarding diabetes in The Gambia as low knowledge has been observed. Programs aimed to improve diabetes knowledge should consider regional and area of residence variations in their designs. The use of mass media and strengthening the education sector in The Gambia may be of importance in raising diabetes knowledge among Gambian adults.

## Background

Diabetes is a chronic metabolic disease that is characterized by elevated levels of blood glucose (or blood sugar) which may, over time, lead to serious damage of the heart, blood vessels, eyes, kidneys, and nerves [1]. Diabetes prevalence is rapidly increasing worldwide affecting approximately 463 million people most of which, about 80%, are from low- and middle-income countries (LMICs) [2, 3]. LMICs are mostly affected due to current rapid demographic transitions from traditional to more urbanized lifestyles [4].

Non-Communicable Diseases (NCDs) in The Gambia have been on the rise. For example, increasing trends in morbidity (19.8%), hospitalization (9.9%), and mortality (23.4%) due to NCDs were observed between 2008 and 2011 [5]. According to The Gambia’s 2018 NCDs national profile, NCDs (including diabetes) account for 34% of all deaths [6], an increase from the 32% reported in 2014 [7]. The World Health Organisation (WHO) predicts that about 4% of The Gambian population could be diabetic by 2030 [8], underscoring the importance of strengthening awareness of NCDs, specifically diabetes, in the country.

Several studies have reported modifiable lifestyle-related risk factors (diet, lack of physical activity) for diabetes [9, 10]. Identification of diabetes risk factors is critical for disease prevention. Increasing disease knowledge and the associated risk factors have significant effects on health behaviour. Self-care behaviours are largely dependent on adequate disease knowledge [11]. For example, having better knowledge about vector-borne diseases in Guyana resulted in increased uptake of preventive measures for malaria and dengue [12]. In China, the level of knowledge regarding chronic obstructive pulmonary disease correlated with the level of self-management behaviour [13]. Additionally, having knowledge about signs and symptoms of a disease is critical for early detection, hence helping with effective management of the condition. It is, therefore, important to understand levels of diabetes knowledge to help come up with appropriate interventions aimed at increasing prevention and appropriate diabetes management. However, most studies regarding diabetes knowledge have focused on patients and health personnel [14, 15]. As an examples, a 2013 study from The Gambia assessed awareness of diabetes mellitus among 200 diabetes patients [16]. However, the study results could not be generalized to the entire population as it focused on patients attending a health facility. Population-based studies examining diabetes knowledge are scant particularly in Sub-Saharan population.

We, therefore, examined self-reported diabetes knowledge (risk factors, and signs and symptoms) among Gambian adults using the 2019–2020 demographic and health survey (GDHS) nationally representative data.

## Methodology

### Study design and data source

This was a cross-sectional study that used secondary data from the 2019–2020 GDHS [17]. The 2019–2020 GDHS is the second DHS conducted in The Gambia, a follow-on to the 2013 survey.

The 2019–2020 GDHS used the updated version of the 2013 Gambia Population and Housing Census as a sampling frame. A two-stage stratified sample was selected. Enumeration areas (EAs) were selected in the first stage using probability proportional to size within each sampling stratum. EAs represented a group of small settlements or a part of a large settlement. There were about 4098 EAs with an average size of 68 households per EA. The second stage involved systematic sampling of households from the selected EAs. A total of 7025 households were selected. All women were aged 15–49 years who were permanent residents of the selected households or visitors who stayed in the selected households the night before the survey were eligible to be interviewed. Further, men aged 16–59 years in half of the selected households were also eligible to be interviewed. In total, 11,865 women and 4636 men were interviewed representing a 95.1% and 86.9% response rate. Approximately 6186 women and 4636 men were asked questions regarding diabetes knowledge.

Questionnaires were used to collect the data. The questionnaires were in English, and the interviewers took part in the translation of the questions into the appropriate local language. The questionnaire was pre-tested from 27 August to 21 September 2019, and actual data collection was between 21 November 2019 and 30 March 2020. All field staff were trained to ensure quality data collection.

### Study variables

#### Outcome variable

The study considered two outcome variables to assess the participants’ knowledge regarding diabetes.

First, knowledge of risk factors for diabetes were examined using eight factors. The following question was asked: “in your opinion, what can increase the risk of having high blood sugar or diabetes?”. Responses included overweight/obese, tobacco use, too much sugar, unhealthy diet, drinking alcohol, family history, and genetics, age. Participants who were able to identify at least three risk factors were categorized as “yes” (i.e., having knowledge on diabetes risk factors) otherwise they were categorized as “no” (i.e., having inadequate knowledge regarding risk factors for diabetes).

Second, knowledge about signs and symptoms for diabetes was assessed using the question: “what are the signs and symptoms of high blood sugar or diabetes?” Responses included fatigue, blurry vision, increased urination, increased thirst, increased hunger, numbness/tingling/burning of feet/hands, weight loss. Participants who were able to identify at least three signs and symptoms were categorized as “yes” (i.e., having adequate knowledge about diabetes signs and symptoms) otherwise they were categorized as “no” (i.e., having inadequate knowledge).

#### Independent variables

We included the following sociodemographic variables in our study namely, age of the participant in years (15–24, 25–34, and ≥ 35), educational level (no formal education, primary, and secondary and higher education), employment (yes/no), religion (Islam and Christianity and other), marital status (never in the union, currently the in union, formerly in the union), region (Banjul, Brikama, Mansakonko, Kerewan, Kuntaur, Janjabureh), residence (rural, urban). Wealth was calculated using household items such as bicycles and principal component analysis was used to create scores, and these were further divided into quintiles forming five groups (richest, rich, middle, poor, and poorest). For the purposes of this study, we combined richest and rich into rich, poorest, and poor into poor, and middle remained the same to create a three-level wealth variable. Access to media was assessed by asking the participants whether they listened to the radio, watch television, or the read newspapers. Those that reported listening to radio or watching television or reading the newspaper at least once a week were regarded as exposed to the media. We further assessed whether the participant has been told by a doctor or health professional that they have high blood sugar (Yes/No).

### Statistical analyses

All the analyses were performed using Stata version 17.0 (Stata Corp LP, College Station, TX). Since it has been reported elsewhere that there are gender differences in health literacy [18–20], we stratified our analysis by gender. Chi-Square tests were performed to examine the differences between selected sociodemographic factors and diabetes knowledge using the “*svy*” command to account for clustering effects and sample weights. To assess the relationship between the selected factors and the outcome variables, a generalized estimating equation (GEE) model was used. Variables with a *p*-value < 0.25 in univariate analysis were considered in the multivariable models. The GEE model was selected to adjust for correlated responses within the dataset since the GDHS data is hierarchical. We also used the sampling weights in the GEE models to account for the survey design. The associations were presented as adjusted odds ratios (AOR) and 95% confidence intervals (CI). We conducted a sensitivity analysis to compare results when participants that reported having diabetes were excluded or included in the analysis. We controlled for diabetes status (yes/no) in the model that included participants with diabetes. Statistical significance was set at *p* < 0.05.

### Ethical considerations

The protocols for survey methodology and all related instruments were approved by institutional review boards (IRBs) at ICF and The Gambia Government/Medical Research Council Joint Ethics Committee in The Gambia. Both IRBs approved the protocols before the commencement of data collection activities. Informed consent was obtained from the study participant before recruiting them into the survey. Ethical standards were applied throughout this GDHS as per Gambia’s ethics committee guidelines and regulations.

## Results

### Knowledge of diabetes risk factors and signs and symptoms

**Fig. 1:**
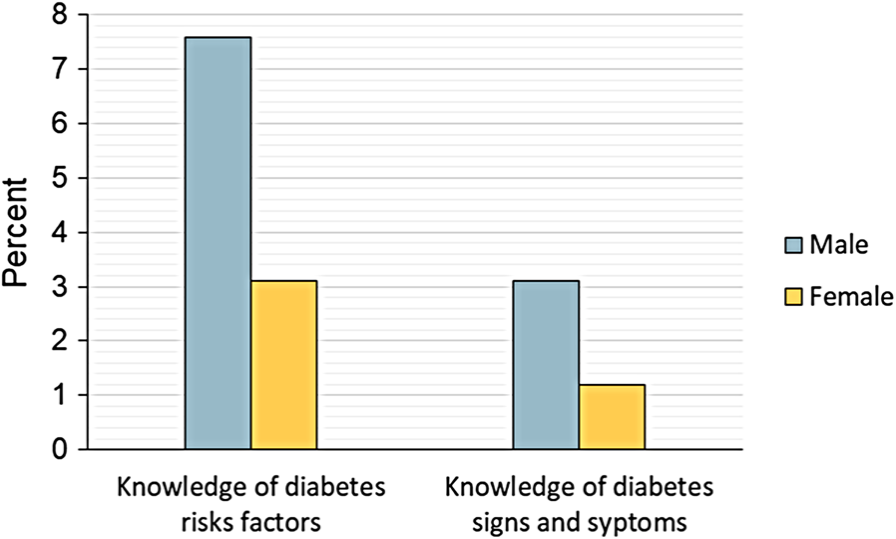
Prevalence of diabetes knowledge by sex

A total of 4636 men and 6186 women were analyzed. Approximately 7.6% and 3.1% of the men had knowledge about diabetes risk factors, and signs and symptoms, respectively. Lower knowledge rates were observed in women with 3.1% having knowledge about diabetes risk factors and 1.2% having knowledge of diabetes signs and symptoms (Fig. 1).

### Distribution of study characteristics according to diabetes knowledge (risk factors)

Table 1 further displays the distribution of participants according to having knowledge of diabetes risk factors. Among men, the difference between those with and without knowledge of diabetes risk factors were significant (*p* < 0.05) in all the variables considered in this study. However, among women, the difference between those with and without knowledge of diabetes risk factors were significant (*p* < 0.05) in terms of educational level, employment status, religion, region, and residence. Specifically, among women that had knowledge of diabetes risk factors, a high proportion had secondary and higher educational levels (66.0%), were Islamic (91.6%), and were living in the urban areas (57.0%).

**Table 1:** Distribution of study characteristics according to knowledge of diabetes risk factors and signs and symptoms

Variables	Men						Women					
	Knowledge of diabetes risk factors			Diabetes signs and symptoms knowledge			Knowledge of diabetes risk factors			Diabetes signs and symptoms knowledge		
	No (n = 4285)	Yes (n = 351)	*p-*value^a^	No (n = 4495)	Yes (n = 141)	*p-*value^a^	n (%) (n = 5996)	n (%) (n = 190)	*p-*value^a^	No (n = 6115)	Yes (n = 71)	*p-*value^a^
Age (years)			** < 0.001**			** < 0.001**			0.235			0.130
15–24	1816 (42.4)	83 (23.5)		1873 (41.7)	25 (18.0)		2447 (40.8)	61 (32.1)		2491 (40.7)	17 (24.0)	
25–34	1048 (24.4)	110 (31.4)		1121 (24.9)	37 (26.5)		1947 (32.5)	74 (39.1)		1992 (32.6)	28 (40.1)	
≥ 35	1421 (33.2)	158 (45.1)		1501 (33.4)	79 (55.6)		1603 (26.7)	55 (28.8)		1632 (26.7)	26 (35.9)	
Educational level			**0.002**			**0.006**			** < 0.001**			** < 0.001**
No formal education	1023 (23.9)	70 (20.0)		1064 (23.7)	29 (20.5)		2097 (35.0)	39 (20.2)		2127 (34.8)	8 (11.6)	
Primary	720 (16.8)	33 (9.4)		7445(16.5)	9 (6.5)		956 (16.0)	26 (13.8)		975 (15.9)	8 (11.6)	
Secondary and higher	2542 (59.3)	248 (70.6)		2686 (59.8)	103 (72.8)		2943 (49.0)	125 (66.0)		3013 (49.3	54 (76.8)	
Wealth			**0.003**			0.005			0.534			**0.008**
Poor	1462 (34.1)	76 (21.6)		1505 (33.5)	33 (22.8)		1998 (33.3)	65 (34.3)		2048 (33.5)	15 (21.9)	
Middle	852 (19.9)	70 (20.0)		896 (19.9)	26 (18.9)		1218 (20.3)	30 (15.4)		1238 (20.2)	9 (12.5)	
Rich	1971 (46.0)	205 (58.4)		2093 (46.6)	82 (58.3)		2780 (46.4)	95 (50.3)		2829 (46.3)	47 (66.4)	
Employment			**0.025**			0.158			**0.001**			0.082
No	1023 (23.9)	62 (17.5)		1061 (23.6)	24 (17.0)		2997 (50.0)	70 (36.9)		3041 (49.7)	26 (37.1)	
Yes	3262 (76.1)	289 (82.5)		3434 (76.4)	117 (83.0)		2999 (50.0)	120 (63.1)		3074 (50.3)	45 (62.9)	
Religion			** < 0.001**			0.394			**0.009**			0.315
Islam	4150 (96.9)	318 (90.7)		4335 (96.5)	133 (94.1)		5817 (97.0)	174 (91.6)		5924 (96.9)	66 (93.5)	
Christianity/other	135 (3.1)	33 (9.3)		160 (3.5)	8 (5.9)		179 (3.0)	16 (8.4)		191 (3.1)	5 (6.5)	
Marital status			** < 0.001**			** < 0.001**			0.624			0.931
Never in union	2402 (56.1)	154 (43.8)		2511 (55.9)	44 (31.5)		1850 (30.9)	65 (34.3)		1895 (31.0)	20 (28.4)	
Currently in union	1823 (42.6)	183 (52.2)		1913 (42.5)	93 (66.0)		3822 (63.7)	116 (61.1)		3890 (63.6)	47 (66.5)	
Formerly in union	60 (1.4)	14 (4.0)		71 (1.6)	4 (2.5)		324 (5.4)	9 (4.6)		330 (5.4)	4 (5.1)	
Access to media			** < 0.001**			** < 0.001**			0.917			0.227
No	564 (13.2)	18 (5.1)		577 (12.8)	6 (4.3)		1673 (27.9)	52 (27.3)		1711 (28.0)	14 (20.0)	
Yes	3720 (86.8)	333 (94.9)		3918 (87.2)	135 (95.7)		4323 (72.1)	138 (72.7)		4044 (72.0)	57 (80.0)	
Region			** < 0.001**			** < 0.001**			** < 0.001**			**0.019**
Banjul	80 (1.9)	9 (2.6)		88 (2.0)	1 (1.2)		80 (1.3)	6 (3.1)		84 (1.4)	2 (3.1)	
Kanifing	971 (22.7)	153 (43.7)		1065 (23.7)	59 (42.1)		1330 (22.2)	63 (33.1)		1370 (22.4)	23 (32.0)	
Brikama	2005 (46.8)	121 (34.4)		2079 (46.3)	47 (33.3)		2666 (44.5)	70 (36.9)		2709 (44.3)	27 (38.4)	
Mansakonko	139 (3.2)	12 (3.3)		148 (3.3)	3 (1.7)		213 (3.5)	17 (9.1)		223 (3.7)	7 (9.9)	
Kerewan	373 (8.7)	16 (4.5)		380 (3.5)	9 (6.6)		549 (9.2)	24 (12.8)		569 (9.3)	4 (5.1)	
Kuntaur	152 (3.5)	11 (3.0)		159 (3.5)	4 (2.3)		261 (4.3)	2 (1.1)		262 (4.3)	1 (1.9)	
Janjanbureh	202 (4.7)	24 (7.0)		211 (4.7)	15 (11.2)		304 (5.1)	3 (1.6)		305 (5.0)	2 (2.0)	
Basse	363 (8.5)	5 (1.5)		365 (8.1)	3 (1.7)		593 (9.9)	5 (2.5)		593 (9.7)	5 (7.6)	
Residence			** < 0.001**			0.087			** < 0.001**			0.902
Rural	1020 (23.8)	36 (10.3)		1034 (23.0)	22 (15.3)		1537 (25.6)	82 (43.0)		1601 (26.2))	18 (25.4)	
Urban	3265 (76.2)	315 (89.7)		3460 (77.0)	119 (84.7)		4459 (74.4)	108 (57.0)		4514 (73.8	53 (74.6)	
Diabetic			** < 0.001**			** < 0.001**			0.086			** < 0.001**
No	4258 (99.4)	341 (97.1)		4464 (99.3)	134 (94.9)		5922 (98.8)	183 (96.6)		6044 (98.8)	61 (85.8)	
Yes	27 (0.6)	10 (2.9)		30 (0.7)	7 (5.1)		74 (1.2)	7 (3.4)		71 (1.2)	10 (14.2)	

Bold values denote statistical significance at the p<0.05 level

^a^*p*-value from Chi-square test

### Distribution of study characteristics according to diabetes knowledge (signs and symptoms)

Among men who had knowledge of diabetes signs and symptoms, a high proportion were aged ≥ 35 years (55.6%), had secondary and higher education (72.8%), resided in rich households (58.3%), were currently in union (66.0%), had media exposure (95.7%), and were dwellers of Kanifing (42.1%) and Brikama (33.3%) regions (Table 1). Significant (*p* < 0.05) differences were observed among women with and without knowledge of diabetes signs and symptoms in terms of educational level, wealth, and region (Table 1).

### Factors associated with diabetes knowledge (risk factors)

Sensitivity analyses for multivariable models revealed that comparing the results from the model that excluded people who reported having diabetes with the model that included diabetic participants (while controlling for diabetes status) yielded consistent results hence results presented included all participants regardless of diabetes status.

Table 2 revealed that compared with men aged 15–24 years, those aged 25–34 years (AOR = 1.84, 95% CI = 1.20–2.82) and ≥ 35 years (AOR = 1.90, 95% CI = 1.12–3.22) were more likely to have knowledge regarding diabetes risk factors. Further, compared with those belonging to Islam, men who belonged to Christianity and other religions were more likely to have knowledge regarding diabetes risk factors (AOR = 2.14; 95% CI = 1.21–3.80). Men with access to the media had 61% increased chances of having knowledge regarding diabetes risk factors compared with those with no access to the media (AOR = 1.61; 95% CI = 1.09–2.37). Regional variations were observed with those dwelling in the Kuntaur and Janjanbureh regions being more likely to have diabetes risk factors knowledge while those from Basse were less like to have diabetes risk factors knowledge compared with those from Banjul. Urban men and those that were diabetic were more likely to have knowledge about diabetes risk factors compared with rural women and those who had no diabetes, respectively.

**Table 2:** Determinants of knowledge of diabetes among Gambian Men and women

Variable	Men				Women			
	Knowledge of diabetes risk factors		Diabetes signs and symptoms knowledge		Knowledge of diabetes risk factors		Diabetes signs and symptoms knowledge	
	AOR (95% CI)	*p*-value	AOR (95% CI)	*p*-value	AOR (95% CI)	*p*-value	AOR (95% CI)	*p*-value
**Age (years)**								
15–24	1.00		1.00				1.00	
25–34	**1.84 (1.20**–**2.82)**	**0.005**	1.94 (0.98–3.85)	0.056			1.99 (0.76–5.17)	0.085
≥ 35	**1.90 (1.12**–**3.22)**	**0.018**	**2.59 (1.08**–**6.17)**	**0.032**			**2.86 (1.15**–**7.13)**	**0.024**
**Educational level**								
No formal education	1.00		1.00		1.00		1.00	
Primary	0.88 (0.54–1.45)	0.623	0.77 (0.32–1.72)	0.547	1.49 (0.87–2.56)	0.147	2.30 (0.94–5.61)	0.068
Secondary and higher	1.21 (0.85–1.72)	0.292	**1.68 (1.08**–**2.60)**	**0.022**	**2.06 (1.46**–**2.92)**	** < 0.001**	**5.59 (2.94**–**10.64)**	** < 0.001**
**Wealth**								
Poor	1.00		1.00				1.00	
Middle	1.05 (0.64–1.71)	0.851	1.20 (0.58–2.48)	0.618			0.88 (0.36–2.30)	0.792
Rich	1.21 (0.73–1.99)	0.466	1.72 (0.88–3.35)	0.111			1.45 (0.50–4.28)	0.499
**Employment**								
No	1.00		1.00		1.00		1.00	
Yes	0.99 (0.67–1.46)	0.974	0.86 (0.46–1.62)	0.644	**1.91 (1.12**–**2.58)**	** < 0.001**	1.18 (0.65–2.23)	0.550
**Religion**								
Islam	1.00				1.00			
Christianity/other	**2.14 (1.21**–**3.80)**	**0.009**			2.40 (0.87–6.66)	0.092		
**Marital status**								
Never in union	1.00		1.00					
Currently in union	1.24 (0.77–1.98)	0.375	**1.91 (1.06**–**3.44)**	**0.031**				
Formerly in union	1.87 (0.86–4.09)	0.115	1.22 (0.34–4.35)	0.759				
**Access to media**								
No	1.00		1.00				1.00	
Yes	**1.61 (1.09**–**2.37)**	**0.017**	**2.04 (1.07**–**3.88)**	**0.030**			1.06 (0.50–2.27)	0.875
**Region**								
Banjul	1.00		1.00		1.00		1.00	
Kanifing	1.48 (0.68–3.17)	0.319	2.48 (0.97–6.31)	0.057	0.64 (0.31–1.35)	0.243	0.58 (0.25–1.34)	0.200
Brikama	0.74 (0.37–1.48)	0.397	1.42 (0.61–3.28)	0.415	**0.23 (0.11**–**0.49)**	** < 0.001**	0.40 (0.16–0.96)	0.041
Mansakonko	2.20 (0.97–4.99)	0.59	2.04 (0.71–5.89)	0.187	**0.09 (0.03**–**0.053)**	**0.001**	2.30 (0.16–6.79)	0.853
Kerewan	1.08 (0.37–3.13)	0.881	2.59 (0.80–8.36)	0.111	**0.06 (0.02**–**0.26)**	** < 0.001**	0.26 (0.04–1.79)	0.173
Kuntaur	**2.87 (1.12**–**7.39)**	**0.029**	3.43 (0.94–12.57)	0.062	**0.01 (0.00**–**0.53)**	** < 0.001**	0.30 (0.03–2.87)	0.299
Janjanbureh	**4.64 (1.71**–**12.65)**	**0.003**	**12.16 (4.42 – 33.49)**	** < 0.001**	**0.01 (0.00**–**0.07)**	** < 0.001**	0.25 (0.03–1.95)	0.184
Basse	**0.34 (0.12**–**0.96)**	**0.042**	0.54 (0.15–1.91)	0.337	**0.01 (0.00**–**0.08)**	** < 0.001**	0.83 (0.29–2.35)	0.445
**Residence**								
Rural	1.00		1.00		1.00		1.00	
Urban	**3.86 (1.99**–**7.48)**	** < 0.001**	1.84 (0.84–4.03)	0.125	**0.05 (0.02**–**0.13)**	** < 0.001**	0.25 (0.05–1.27)	0.094
**Diabetic**								
No	1.00		1.00		1.00		1.00	
Yes	**4.20 (1.49**–**11.82)**	**0.006**	**5.78 (1.45**–**22.91)**	**0.012**	3.09 (0.81–11.75)	0.099	**9.39 (3.09**–**28.58)**	** < 0.001**

Bold values denote statistical significance at the p<0.05 level

*AOR* adjusted odds ratio, *CI* confidence interval

Among women, those with secondary and higher education (AOR = 2.06, 95% CI = 1.46–2.92), and employed (AOR = 1.91, 95% CI = 1.12–2.58), were more likely to have knowledge of diabetes risk factors as compared with those with no formal education and unemployed, respectively. Compared with women from the Banjul region, those from Brikama, Mansakonko, Kerewan, Kuntaur, Janjanbureh, and Basse were less likely to have knowledge regarding diabetes risk factors. Urban women were also less likely to have knowledge about diabetes risk factors compared with rural women (Table 2).

### Factors associated with diabetes knowledge (signs and symptoms)

Among men, those aged ≥ 35 years (AOR = 2.59, 95% CI = 1.08–6.17), with secondary and higher education (AOR = 1.68, 95% CI = 1.08–2.60),in union (AOR = 1.91, 95% CI = 1.06–3.44), having access to media (AOR = 2.04, 95% CI = 1.07–3.88) and diabetic (AOR = 5.78, 95% CI = 1.45–22.91) were more likely to have knowledge regarding diabetes signs and symptoms compared with those aged 15–24 years, with no form education, never in the union, those that had no access to the media, and non-diabetic, respectively. Men from Janjanbureh (AOR = 12.16, 95% CI = 4.42–33.49) were more likely to have knowledge about diabetes signs and symptoms compared with those from Banjul albeit with a wide confidence interval (Table 2).

Older women (aged ≥ 35) (AOR = 2.86, 95% CI = 1.15–7.12), with secondary and higher education (AOR = 5.59, 95% CI = 2.94–10.64), and who were diabetic (AOR = 9.39, 95% CI = 3.09–28.58) were more likely to have knowledge about diabetes signs and symptoms compared with younger women (15–24 years), with no formal education, from poor households, and those with no diabetes (Table 2).

## Discussion

This is the first nation-wide study to assess knowledge of diabetes among Gambian adults and the associated factors. Our findings revealed that sociodemographic factors such as the age of participants and educational level were associated with knowledge regarding diabetes. Specifically, among men, older age was associated with 84% (for those aged 25–34 years) and 90% (for those aged ≥ 35 years) increase in the likelihood of having knowledge regarding diabetes risk factors compared with younger age (15–24 years) while the attainment of secondary and higher education was associated with 68% increase in the likelihood of knowing signs and symptoms of diabetes. Among women, a 2.06 and 5.59 increase in the odds of having appropriate knowledge regarding diabetes risk factors, and signs and symptoms, respectively, was observed among those with a secondary and higher education compared with those with no formal education.

Among men, knowledge regarding diabetes risk factors, and signs and symptoms was 7.6% and 3.1%, respectively. About 3.1% of the women had knowledge about diabetes risk factors while 1.2% had knowledge about diabetes signs and symptoms. Diabetes knowledge was relatively lower in the Gambia compared with knowledge levels reported in Debre Berhan town in Ethiopia where 56.0% had good knowledge regarding diabetes [21]. The huge difference in the prevalence could be attributed to the different measurements and definitions of diabetes risk factors and how these were measured in the questionnaire. For example, while in the current study knowledge was measured by allowing participants to mention at least three correct responses to the risk factors or signs and symptoms, the Ethiopian study only requested participants to state whether they know diabetes risk factors (yes or no) [21]. Nevertheless, our study revealed relatively low knowledge regarding diabetes among Gambian adults and more needs to be done to raise awareness about the disease, the causes, and preventive measures.

Gambian adults who were older were more likely to have knowledge regarding diabetes compared with younger Gambians. In Saudi Arabia, adults aged ≥ 35 years had better knowledge about diabetes [22]. One of the reasons for this observation is that diabetes is more prevalent in the older population compared with the younger population [23]. However, it is important to adopt lifestyles that may help prevent diabetes at an early stage and thus, awareness programs targeting younger populations on diabetes prevention through lifestyle changes such as frequent physical activities and adoption of healthy diets are important.

Attainment of secondary and higher education was associated with increased likelihood of having knowledge regarding diabetes among both men and women from The Gambia. This is consistent with a study conducted in Melbourne, Australia, and Galle district in Sri Lanka where attaining a higher educational level was associated with better diabetes knowledge among patients with Type 2 diabetes [24, 25]. It has been reported that education can lead to accuracy in health beliefs and knowledge, may improve critical skills (such as literacy and cognitive ability), and therefore, may lead to better lifestyle choices and improved comprehension of health messages [26]. Educated people are more likely to learn about health behaviors and understand their health needs. Further, education attainment can create opportunities such as better incomes, through employment, that may help them achieve better health.

Access to the media was associated with better diabetes knowledge among Gambian men. The role of the media in raising awareness of NCDs such as diabetes cannot be overlooked. Media presents an opportunity for health professionals to convey messages on diabetes prevention and management [27]. It is, therefore, important to design awareness campaigns with the use of the media as a way through which to pass important preventive messages. We observed heterogeneities regarding diabetes knowledge among different regions and areas of residence (urban/rural) underscoring the need for tailored NCD education (especially diabetes) for people living in different areas in The Gambia.

Those that reported having been told by a medical doctor or health professional that they had high blood sugar were more likely to have diabetes knowledge of risk factors and symptoms among both men and women compared to those who were non-diabetic. Those that have been diagnosed with diabetes are likely to undergo counselling by the medical practitioners and hence are more likely to have correct knowledge. Additionally, diabetic participants are more likely to have the interest to know more about their condition compared with non-diabetic individuals as such, they may possess a better understanding about the condition. Those whose family members suffer from diabetes are also likely to possess better knowledge of the condition as they may develop an interest to understand how the condition may be prevented and managed [28].

### Future implications

As NCDs become increasingly important in developing countries like the Gambia, effective strategies to improve knowledge about the disease will be critical. Therefore, this study provides the following future implications. First, the low prevalence of knowledge of diabetes highlights the urgent need to come up with programs that will raise awareness about the condition in the Gambia. Second, the factors identified in this current analysis, for example, the demographic factors, are key to designing and implementing effective programs for raising diabetes awareness in the Gambia. Third, future studies may wish to employ intervention study designs where baseline knowledge may be assessed and an educational programme regarding diabetes be designed as an intervention to check the effectiveness of the programme in increasing diabetes knowledge.

### Strengths and limitations

The study used nationally representative data hence strengthening the generalizability of the results. This is the first population-based study to examine diabetes knowledge in The Gambia and as such, it may serve to inform policymakers and public health program managers on the urgent need to raise awareness and formulate context-specific awareness programs targeting specific subgroups in the population. The study has limitations. First, the cross-sectional design of the survey meant that we could not infer causality of all the associations observed in the current analysis. Second, some of our estimates had wide confidence intervals possibly due to smaller samples in some categories hence the results should be interpreted with caution. Third, the study relied on the data that was collected from respondents’ self-reported knowledge regarding the risk factors, signs, and symptoms of diabetes, thus, our results are prone to recall bias. Fourth, since this was secondary data analysis, we were restricted to consider variables present in the dataset as such, other important variables like lifestyle factors were not analyzed because they were not collected.

## Conclusion

Our results have revealed a low prevalence of diabetes knowledge among Gambian adults. Efforts need to be made to raise awareness about NCDs including diabetes as these are emerging conditions in developing countries like the Gambia. Attention needs to be placed on different high-risk groups identified in the current study to improve their diabetes knowledge. This may, in turn, help them adopt appropriate lifestyles that may help prevent diabetes. The study revealed the important role played by education in increasing the likelihood of having knowledge regarding diabetes. Therefore, improving the education sector may play a role in improving health literacy including knowledge about NCD (particularly diabetes) in the Gambia.

## Data Availability

The data that support the findings of this study are available online from the DHS measure and approval to access can be obtained the DHS Measure.

## Abbreviations

AOR: Adjusted odds ratios
CI: Confidence interval
EA: Enumeration area
GDHS: Gambia Demographic Health Survey
GEE: Generalized Estimation Equation
IRB: Institution Review Board
LMICs: Low- and Middle-Income Countries
NCD: Non-Communicable Disease
WHO: World Health Organization
